# Visualization of BOK pores independent of BAX and BAK reveals a similar mechanism with differing regulation

**DOI:** 10.1038/s41418-022-01078-w

**Published:** 2022-10-26

**Authors:** Raed Shalaby, Arzoo Diwan, Hector Flores-Romero, Vanessa Hertlein, Ana J. Garcia-Saez

**Affiliations:** 1 Institute for Genetics and Cologne Excellence Cluster on Cellular Stress Responses in Aging-Associated Diseases (CECAD), University of Cologne, Joseph-Stelzmann-Straße 26, 50931 Cologne, Germany; 2 Interfaculty Institute of Biochemistry, Eberhard-Karls-Universität Tübingen, Tübingen, Germany

**Keywords:** Cell biology, Biochemistry

## Abstract

BOK is a poorly understood member of the BCL-2 family of proteins that has been proposed to function as a pro-apoptotic, BAX-like effector. However, the molecular mechanism and structural properties of BOK pores remain enigmatic. Here, we show that the thermal stability and pore activity of BOK depends on the presence of its C-terminus as well as on the mitochondrial lipid cardiolipin. We directly visualized BOK pores in liposomes by electron microscopy, which appeared similar to those induced by BAX, in line with comparable oligomerization properties quantified by single molecule imaging. In addition, super-resolution STED imaging revealed that BOK organized into dots and ring-shaped assemblies in apoptotic mitochondria, also reminiscent of those found for BAX and BAK. Yet, unlike BAX and BAK, the apoptotic activity of BOK was limited by partial mitochondrial localization and was independent of and unaffected by other BCL-2 proteins. These results suggest that, while BOK activity is kept in check by subcellular localization instead of interaction with BCL-2 family members, the resulting pores are structurally similar to those of BAX and BAK.

## Bullet points

- BOK-induced pores were directly visualized in liposomes using negative staining EM.
- BOK apoptotic activity is comparable to and independent of BAX and BAK, including formation of rings in apoptotic mitochondria.
- Apoptosis induction by BOK is limited by partial mitochondrial localization.

## Introduction

The mitochondrial pathway of apoptosis is controlled by the proteins of the BCL-2 family [1]. The effector proteins BAX and BAK directly mediate the key step of permeabilization of the mitochondrial outer membrane (MOM), which releases cytochrome c and Smac into the cytosol to induce the caspases activation leading to cell death [2–4]. The activity of BAX and BAK is counteracted by the prosurvival BCL-2 proteins, like BCL-2, BCL-xL and MCL-1 [5, 6]. The pro-apoptotic BH3-only proteins including tBID, BIM or PUMA, promote cell death by activating the effectors and/or blocking the anti-apoptotic family members [3].

BCL-2 related ovarian killer (BOK) is BCL-2 family member was initially categorized as a pro-apoptotic BCL-2 family member based on sequence similarity with BAX and BAK and on transient overexpression experiments [7]. This view was supported by studies reporting that overexpression of BOK can induce membrane permeabilization and mitochondrial apoptosis, independent of BAX and BAK [8]. The increased severity of the phenotype of BAX^−/−^ BAK^−/−^ BOK^−/−^ mice compared to BAX^−/−^ BAK^−/−^ animals provided additional support for this model [9].

The structure of inactive, monomeric BOK in soluble form presents the typical BCL-2 fold [9, 10], with two central hydrophobic helices surrounded by amphipathic α-helices [11]. The two central helices of BCL-2 effector proteins become embedded in the membrane when the protein is activated, leading to permeabilization [12–14]. The structure of BOK additionally contained an occluded hydrophobic groove that could underlie the inability to interact with the BH3 domain of BH3-only proteins. It has been proposed that BOK can interact with MCL-1 and BFL-1 via its BH3-domain, but not with other antiapoptotic proteins [7, 15]. This is a distinct feature of BOK and suggests alternative regulatory mechanisms. Accordingly, BOK is maintained at low levels by proteasomal degradation, which upon ER stress lead to BOK accumulation and cell death induction [8].

Despite these recent advances, BOK remains an enigmatic protein far less understood than most BCL-2 family members. One key aspect is whether BOK is able to mediate mitochondrial permeabilization directly via the opening of membrane pores. Related to this, little is known about the molecular mechanism underlying the pore activity of BOK and how it compares to that of BAX and BAK, especially regarding the role of protein oligomerization, as well as regulation of the pore activity.

Here, we studied the properties of BOK pores independent of BAX and BAK by combining experiments in chemically controlled systems with model membranes and in cells. We directly visualize BOK nanoscale assemblies and membrane pores, which presented similar properties to those of BAX and BAK, in line with comparable oligomerization properties. However, in contrast to BAX and BAK, the apoptotic activity of BOK was lower compared to them and was not affected by other pro- and anti-apoptotic BCL-2 proteins. Our data indicate that BOK can directly act as a BCL-2 effector independently of BAX and BAK, albeit with reduced efficiency due to limited mitochondrial localization.

## Results

### The C-terminus of BOK determines thermostability and pore forming activity

**Fig. 1:**
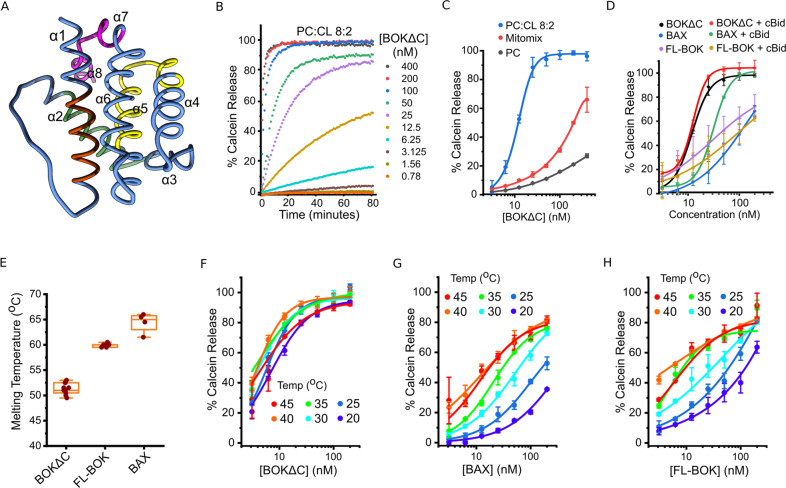
Permeabilizing activity of recombinant BOK in lipid vesicles depends on the C-terminus and the membrane lipid composition. **A** Structure of human BOK∆C (PDB:6CKV) with the BH domains highlighted in different colors; BH1: orange, BH2: green, BH3: yellow and BH4: purple. BOK∆C structure contains 8 alpha-helices forming a typical BCL-2 fold, each individual helix is numbered. **B** The kinetics of calcein release from LUVs with the composition (PC:CL 8:2) induced by different concentrations of BOK∆C. Calcein release was normalized to the maximum release induced by Triton-X100. **C** Percentage of calcein release from LUVs with the lipid compositions: PC, PC:CL 8:2 and mitochondrial mixture (PC:PE:PI:PS:CL 49:27:10:10:4), induced by different concentration of BOK∆C. Calcein release was normalized to the maximum release induced by Triton-X100. **D** Effect of BAX, BOK∆C and FL-BOK recombinant proteins on LUVs (PC:CL 8:2) membrane permeability in the presence and absence of cBID (40 nM) assessed by calcein release and normalized to the maximum release induced by Triton-X100. **E** Estimated melting temperature of BAX, BOK∆C and FL-BOK derived from thermal shift assay experiments using SYPRO Orange dye. **F**, **G**, **H** Permeabilization activity of BAX, BOK∆C and FL-BOK on LUVs (PC:CL 8:2) at different temperatures, measured and calculated like in **B**. **C**–**H** Values correspond to mean ± SD from at least 3 individual experiments.

To investigate the interaction of BOK with membranes, we produced recombinant, monomeric, human BOK lacking the last 24 amino acids corresponding to the C-terminal membrane anchor (hereafter BOK∆C) (Fig. 1A and S1), similar to [10, 16]. We also generated full length BOK (FL-BOK) using a similar strategy to BAX and BCL-xL [17, 18]. We obtained a much lower yield, with FL-BOK eluting as a mixture monomers and dimers (Fig. S1). BOK∆C was then fluorescently tagged using sortase A enzyme with 95% efficiency and 1:1 stoichiometry (Fig. S2 and Materials and methods).

We used liposome permeabilization assays to compare the pore forming activity of FL-BOK and BOK∆C in vitro. By monitoring calcein release from large unilamellar vesicles (LUVs), we found that BOK∆C could efficiently permeabilize liposomes, with a composition containing cardiolipin as a simplistic model of the MOM, in a concentration-dependent manner (Fig. 1B), in agreement with previous work [10, 16]. This indicates that the membrane permeabilizing activity of BOK does not require the transmembrane C-terminal region, even if it plays a role in membrane targeting in cells [19]. The extent of permeabilization correlated with the amount of the negatively charged lipid cardiolipin in the liposomes (Fig. 1C). Cardiolipin is a mitochondria-specific phospholipid containing four acyl chains that induces negative membrane curvature, which may also contribute to pore activity. It has been reported to be a key lipid in pore formation by BCL-2 proteins [16, 20–23], although other negatively charged lipids like phosphatidylglycerol can promote comparable pore activity in vitro [16].

BOK∆C was able to spontaneously relocate to liposomes and to induce their permeabilization at room temperature (Fig. 1B–D), unlike BAX (in full-length form), which required co-incubation with cBID for its activation. Neither for BAK∆C, as previous reports required the use of a histidine tag to drive its association with liposomes doped with nickel [24–26]. These results, together with the low effect of cBID on BOK∆C activation, suggested that recombinant BOK∆C was auto-active under our experimental conditions. However, the membrane permeabilizing activity of FL-BOK (dimer fraction) in vitro was similar to that of BAX alone and significantly lower than that of BOK∆C at room temperature (Fig. 1D). Still, cBID did not have a significant effect on FL-BOK activity, suggesting that it is not as efficient as direct activator as in the case of BAX.

Previous studies have proposed that these differences between BOK∆C and BAX (and BAK) could be attributed to the difference in their thermal stability [10]. To check whether the differences in the pore activity of FL-BOK and BOK∆C could be related to this, we determined the melting temperature of FL-BOK, which to our surprise, was higher than that of BOK∆C and close to that of BAX (Fig. 1E). We then compared the energetic threshold for BOK∆C, FL-BOK and BAX pore activity by quantifying the extent of LUV permeabilization at different temperatures. As shown in (Fig. 1F–H), both BAX and FL-BOK could be activated by increasing the temperature, as previously reported for BAX [27–29]. However, increasing temperature had no effect on BOK∆C.

### Direct visualization of BOK pores in liposomes by electron microscopy

**Fig. 2:**
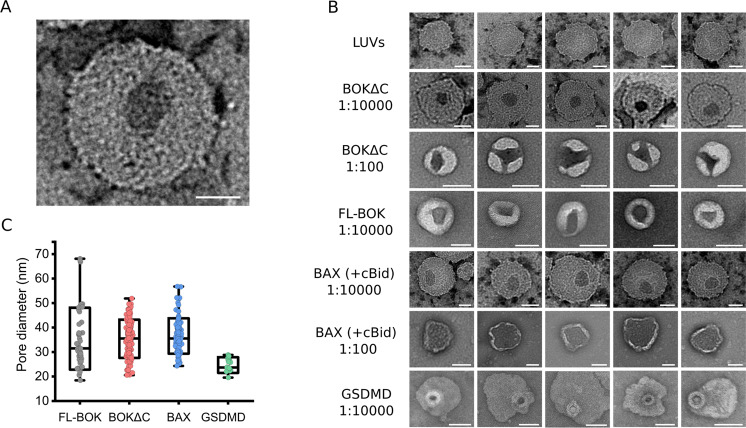
Visualization of BOK pores in liposomes. **A** Representative negative staining EM image of a BOK pore in an LUV with the composition PC:CL 8:2. **B** Representative negative staining EM images from incubation of LUVs with different ratios of BAX, BOK∆C, FL-BOK and GSDM-D (activated by Caspase11). Scale bar for all images in **A**–**B**, 50 nm. **C** Quantification of pore size distribution of BAX, BOK∆C, FL-BOK and GSDM-D incubated with LUVs with protein:lipid molar ratio of 1:10000.

We then aimed at direct imaging of BOK pore formation in liposomes using negative staining electron microscopy (EM). We incubated BOK∆C, FL-BOK, FL-BAX (activated by cBID) and GSDMD (activated by Caspase11) with LUVs for 1 h using 1:100 and of 1:10000 protein:lipid molar ratios. As shown in (Fig. 2A, B), BOK∆C induced liposome alterations depending on protein concentration. Interestingly, almost all permeabilized liposomes had only one pore. At low protein amounts BOK∆C formed relatively well-defined pores with a ring shape, which became more irregular and seemingly associated with membrane invaginations at higher protein:lipid ratio. At lower concentration they adopted a broad distribution of diameters ranging between 20 and 50 nm, with the average around 35 nm (Fig. 2C). With increasing concentration of BOK∆C, the pore diameter increased and often led to complete rupture of the liposomes.

We could also efficiently detect FL-BOK pores in liposomes, which presented a similar shape to those of BOK∆C, although they also seemed to induce membrane invaginations at lower protein:lipid ratio (Fig. 2B). We did not observe a significant difference in the size of pores formed by BOK∆C and FL-BOK, although the distribution of diameters slightly increased for the full-length protein (Fig. 2C).

When compared to other pore forming proteins involved in cell death (Fig. 2B, C), similar results in terms of pore size and shape were obtained for BOK and BAX pores, suggesting that both proteins follow the same mechanisms of membrane permeabilization. Remarkably, the pores formed by GSDM-D were smaller and their size distribution was narrower. In addition, protein density at the pore edge was consistently seen only in the case of GSDM-D. Instead, in the case of BOK and BAX, no protein density could be observed around the pore rims, also in line with previous analysis of BAX pores by EM [13, 30].

### BOK exists as a mixture of oligomeric species in the membrane

**Fig. 3:**
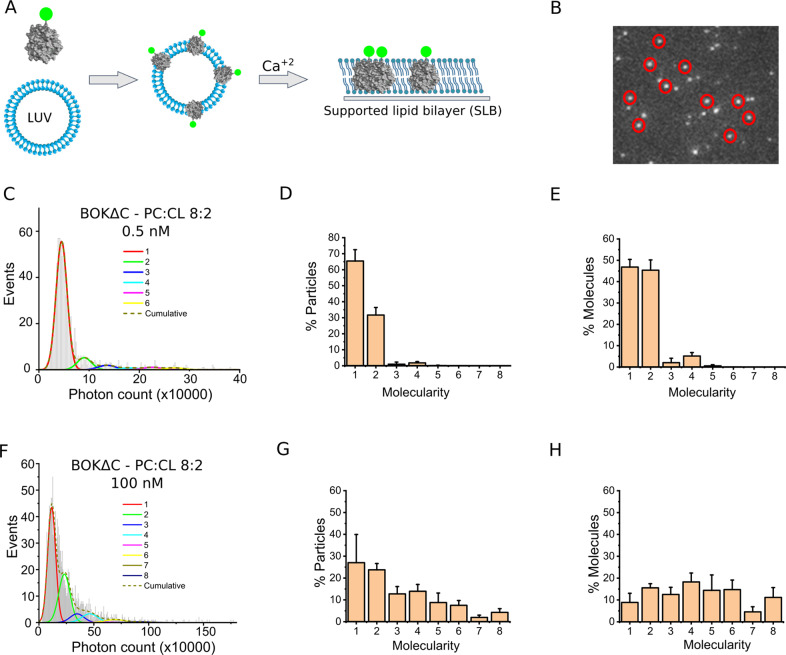
BOK∆C oligomerizes into multiple coexisting species in SLB. **A**, **B** Schematic representation of the single-molecule stoichiometry analysis. (**A**) BOKΔC labelled with Atto488 dye was incubated with the LUVs with the composition PC:CL 8:2, followed by the formation of SLB on coverslip using calcium chloride. **B** Single particles of BOKΔC labelled with Atto488 dye bound to SLB formed from PC:CL 8:2 liposomes resolved with TIRF microscope. **C**–**H** Analysis of Atto488-BOKΔC oligomerization in the membrane. Particle fluorescence intensity distribution from different experiments were fitted with a linear combination of eight Gaussians to estimate the abundance of different molecularities. The cumulative fit is shown as a dashed line. The percentage of each species is derived from the area under each fitted Gaussian. The error bars correspond to the average error for each oligomeric species from three independent experiments with Particles >500 per condition per experiment.

These results suggested that BOK follows a similar mechanism of membrane permeabilization to that of BAX and BAK, for which the formation of lipid/protein pores is accompanied by assembly into multiple oligomeric species [21, 28, 31–33]. To compare the oligomerization properties of BOK in the membrane with those of BAX, we used total internal reflection fluorescence (TIRF) single-molecule imaging as in our previous work [28]. We incubated fluorescently tagged BOK∆C 0.5 nM and 100 nM for 1 h with liposomes, allowing for protein binding and oligomerization [34]. The resulting proteoliposomes were then used to produce supported lipid bilayers (SLBs), which were imaged with TIRF microscopy (Fig. 3A).

The brightness of single BOK∆C-488 particles in the membrane showed a broad distribution typical of multiple, coexisting oligomeric species (Fig. 3B, F). The particle fluorescence intensity distribution for individual BOK∆C-488 particles in SLBs was then fitted to multiple Gaussians to calculate the relative fraction of each species (Fig. 3D, E, G, H). The results obtained showed that, in contrast to BAX and BAK [28, 33, 35], oligomerization took place without activation by BH3-only proteins or heat incubation. The size of the oligomers increased with protein concentration, following a similar trend like BAX but not BAK [35]. While at lower concentrations BOK∆C-488 particles existed as a mixture of mainly monomers and dimers, with increasing protein density in the membrane we detected higher oligomeric forms. Oligomeric species above octamers could not be quantified due to inherent limitations of the technique (see Methods). Under these experimental conditions, less than 10% of BOK∆C-488 particles were in monomeric form. Together, these results indicate that BOK has the ability to autoactivate and to recruit additional BOK molecules to the complex, similar to reports for BAK and BAX [28, 35].

As controls, we confirmed that the particle brightness of BOK∆C-488 showed a narrow distribution corresponding to monomers when it was imaged on glass and that membrane binding and oligomerization were not efficient on SLBs made of PC only (Fig. S3). This suggests that cardiolipin, or a negatively charged lipid, is required for BOK∆C-488 binding and oligomerization in the membrane.

### Apoptosis induction by BOK is comparable to and independent of BAX and BAK

**Fig. 4:**
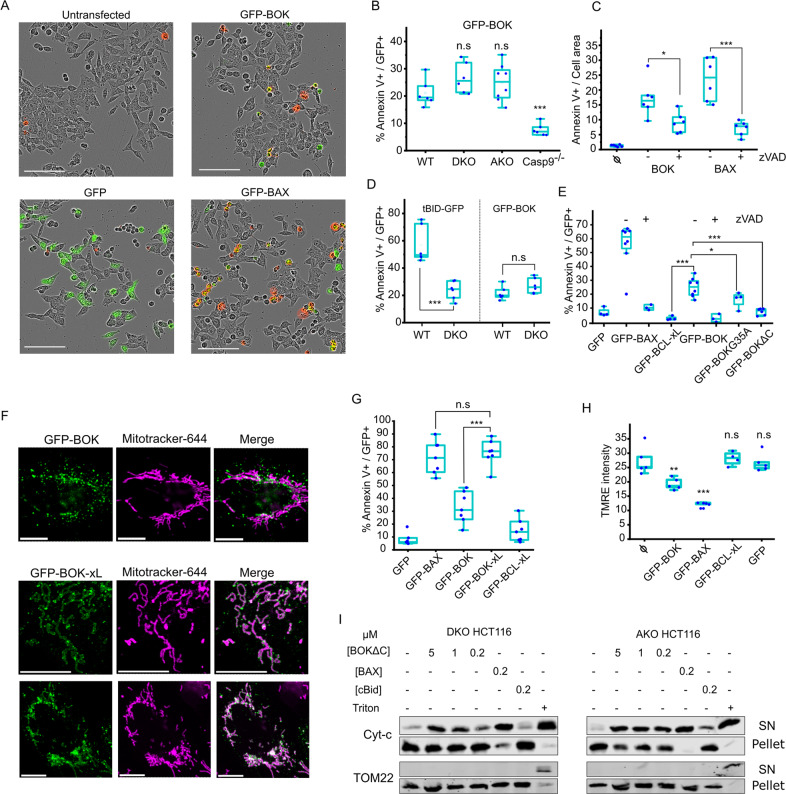
Apoptosis induction by BOK is comparable to and independent of BAX and BAK. **A** Representative images of HCT-AKO cells expressing GFP-BAX, GFP-BOK and GFP 16 h after transfection. Transfected cells and annexin V+ cells appear in green and red respectively. Scale bar; 100 μm. **B** Effect of GFP-BOK overexpression on cell death in different cell lines (HCT-WT, DKO, AKO and Casp9 KO), measured as percentage of cells with Annexin V from the total transfected cells (Annexin V+/GFP+). **C** Effect of BAX and BOK overexpression on cell death in HCT AKO, in the presence or absence of ZVAD, measured as fraction of cells with Annexin V normalized to total cell area. **D** Effect of tBID-GFP and GFP-BOK overexpression on cell death in different cell lines (HCT-WT and DKO) quantified as in **B**. **E** Effect of GFP-BAX, GFP-BOK, GFP-BOK G35A, GFP-BOK∆C, GFP-BCL-xL and GFP overexpression on cell death in HCT AKO, in the presence or absence of ZVAD and calculated as in (**B**). ϕ: untransfected. **F** Representative images of GFP-BOK and GFP-BOK-xL subcellular localization 16 h after transfection in U2OS BAX^−/−^/BAK^−/−^ cells. BOK appears depicted in green and mitochondria (labelled with Mitotracker-644) in magenta. Scale bar, 10 µm. **G** Effect of GFP-BAX, GFP-BOK, GFP-BOK-xL, GFP-BCL-xL and GFP overexpression on cell death in HCT AKO quantified as in **B**. **H** Effect of GFP-BAX, GFP-BOK, GFP-BCL-xL and GFP overexpression on mitochondrial depolarization in U2OS DKO cells, measured as a decrease on TMRE signal. ϕ: untransfected. **I** Release of cytochrome c from isolated mitochondria from HCT116 cells was assessed by immunoblotting of pellet (mitochondria) and supernatant fractions. Antibody against TOM22 was used as a control. ***P ≤ 0.001, **P ≤ 0.01, *P ≤ 0.05, ns not significant. Experiments were averages of at least three replicates.

To study BOK membrane pores in the complex environment of the cell, we analyzed its ability to induce apoptosis in living cells by transiently expressing BOK fused to GFP (GFP-BOK). We used HCT116 lines including wild type (WT HCT116), BAX/BAK double knock out (DKO HCT116), as well as a cell line lacking most BCL-2 proteins in which BOK was additionally knocked out, all BCL-2 knock out (AKO HCT116) [36, 37], which allowed us to evaluate the role of other BCL-2 proteins on the apoptotic activity of BOK. The extent of cell death was assessed by Annexin-V-Alexa647 staining and normalized to transfected (GFP-positive) cells (Fig. 4A).

Remarkably, GFP-BOK efficiently induced apoptotic cell death in AKO HCT116 cells (Fig. 4B), which we also confirmed with overexpression of an untagged version of BOK (Fig. 4C). This clearly demonstrates that BOK can directly mediate mitochondrial permeabilization, thus discarding any requirement for the effectors BAX and BAK, as well as the possibility that BOK killing activity is the result of it inducing a phenotypic switch in the anti-apoptotic BCL-2 members. As reported for BAX and BAK [36], BOK overexpression alone was also sufficient to induce cell death in AKO HCT116 cells, indicating that BH3-only proteins are not necessary for its activation either.

We recently showed that tBID can also mediate mitochondrial permeabilization in absence of BAX and BAK [37]. Indeed, both tBID-GFP and GFP-BOK induced a similar extent of cell death in AKO HCT116 cells. Yet, in contrast to BOK, tBID-induced apoptosis was significantly reduced in DKO HCT116 cells compared to AKO HCT116 cells, as we showed before [37]. This indicates strong inhibition of tBID, but not of BOK, by the anti-apoptotic BCL-2. In agreement with this, the extent of GFP-BOK induced cell death in WT, DKO and AKO HCT116 cell lines was comparable (20-30%). Since both DKO and WT cells harbor anti-apoptotic BCL-2 proteins, these results add to the evidence that the apoptotic activity of BOK is not affected by the antiapoptotic family members (Fig. 4B). Compared to AKO HCT116 cells, overexpression of tBID-GFP led to an increase in cell death in WT HCT116 cells expressing BAX and BAK (Fig. 4D), which underscores the role of tBID as an activator of BAX and BAK. This effect was not observed for GFP-BOK overexpression, suggesting that BOK cannot act as BAX/BAK activator.

Intrinsic instability leading to auto-activation counteracted by proteasomal degradation has been proposed as an mechanism for regulation of BOK apoptotic activity. In agreement with this, the GFP-BOK G35A mutant, with increased protein stability, showed a slight reduction in apoptosis induction in AKO HCT116 cells (Fig. 4E). In contrast, the significantly impaired cytotoxic activity of GFP-BOK∆C highlights the key role of the C-terminal tail on BOK apoptotic function (Fig. 4E).

Remarkably, only a fraction of GFP-BOK localized to mitochondria, raising the question whether this could a reason for the lower apoptotic activity of BOK in cells (Fig. 4F). To test this hypothesis, we produced a chimeric protein with the C-terminal tail of BOK replaced by that of BCL-xL (GFP-BOK-xL), which almost exclusively localized to mitochondria (Fig. 4F). Importantly, GFP-BOK-xL presented enhanced apoptotic activity comparable to BAX (Fig. 4G). These results directly link the lack of effective mitochondrial localization with the limited BOK activity in cells.

The significant reduction in cell death in HCT caspase-9 knock out cells confirmed that BOK-mediated cell death is of apoptotic nature (Fig. 4B). As additional controls, we confirmed that the cytotoxic activities of BAX and BOK were reduced by the caspase inhibitor zVAD (Fig. 4E). Also, cells transfected with either GFP alone or with the anti-apoptotic GFP-BCL-xL showed minimal or insignificant cell death. We further confirmed loss of mitochondrial membrane potential (TMRE intensity) as a proxy for mitochondrial permeabilization in U2OS BAX^−/−^/BAK^−/−^ cells transfected with GFP-BOK (Fig. 4H). Finally, we tested the ability of recombinant BOK∆C to permeabilize isolated mitochondria from DKO HCT116 and AKO HCT116 cells. As shown in (Fig. 4I), BOK∆C released cytochrome c in a concentration dependent manner, with an activity comparable to cBID but significantly lower than recombinant FL-BAX. These results indicate that BOK can directly induce mitochondrial permeabilization independently of BAX, BAK and other BCL-2 proteins.

### BOK forms ring-like structures in apoptotic mitochondria

Previous studies reported that BOK, in contrast to BAX and BAK, mainly localizes to the ER and Golgi [19] and that it accumulates at the contact sites between the ER and mitochondria, where it plays a role in regulating calcium fluxes between the two organelles [38, 39]. This is in agreement with our data in Fig. 4F. Yet, to mediate mitochondrial permeabilization in absence of other BCL-2 proteins, we reasoned that at least a fraction of BOK localized to mitochondria should assemble there into oligomers responsible for the opening of apoptotic pores.

**Fig. 5:**
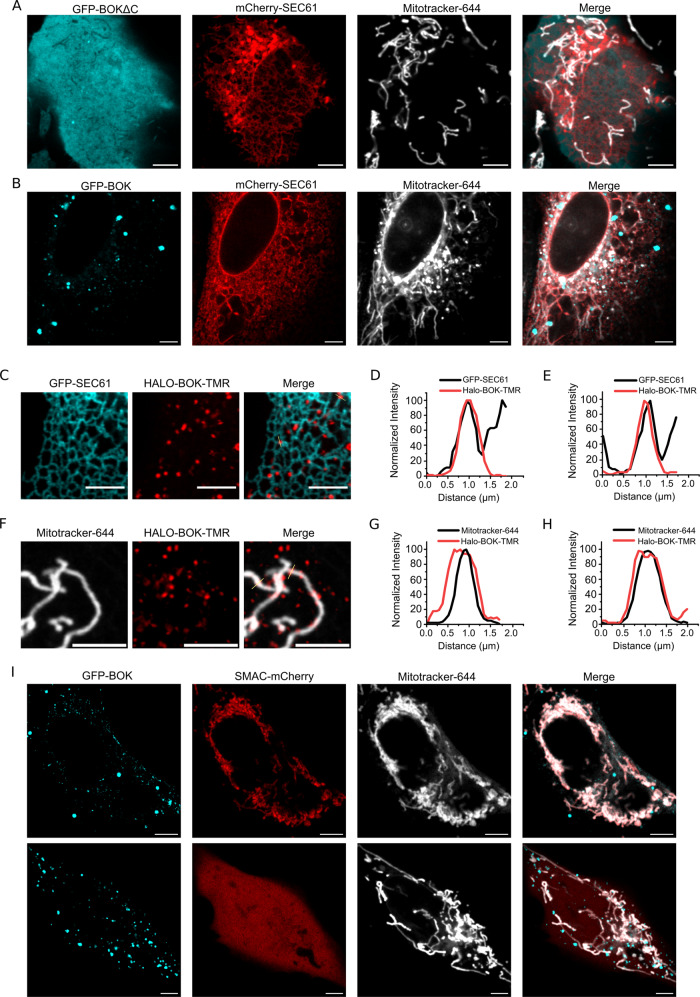
BOK localizes partially to mitochondria and to the ER via its C-terminal anchoring domain. **A**, **B** Representative images of GFPBOK∆C and GFP-BOK subcellular localization 16 h after transfection in U2OS BAX^−/−^/BAK^−/−^ cells. BOK appears depicted in cyan, ER (marked with SEC-61) in red and mitochondria (labelled with Mitotracker-644) in grey. **C**, **F** Representative images of HALO-BOK subcellular localization. BOK appears depicted in red, ER (marked with SEC-61) in cyan and mitochondria (labelled with Mitotracker) in grey. **D**–**H** Line profile of HALO-BOK clusters signal together with the ER (**D**, **E**, SEC61) or mitochondrial signal (**G**, **H**, Mitoctracker-644). Lines are shown in **C**, **F**. Intensity was normalized to 100. **I** Representative images of GFP-BOK subcellular localization and MOMP induction. BOK appears depicted in cyan, Smac-mCherry in red and mitochondria in grey. Scale bar for all images, 5 µm.

To address this question in more detail, we explored the subcellular localization BOK in U2OS BAX^−/−^/BAK^−/−^ cells with respect to mitochondria and/or ER using GFP or Halotag fusion proteins (Fig. 5). In agreement with the lack of apoptotic activity, we found that GFP-BOK∆C presented a diffuse cytosolic distribution, which underscores the key role of the C-terminal anchor for membrane targeting in cells (Fig. 5A). GFP-BOK instead constitutively accumulated in clusters reminiscent of the apoptotic foci formed by BAX and BAK [40, 41] in absence of apoptotic triggers (Fig. 5B). These GFP-BOK clusters presented partial co-localization with mitochondria (dyed with Mitotracker-644) as well as with the ER network (stained with GFP-SEC61), which in some occasions coincided with overlapping points between the two organelles (Fig. 5C–H). 16 h after transfection, only a fraction of the cells expressing BOK had undergone mitochondrial permeabilization measured by Smac release, although the subcellular distribution of BOK was comparable between cells in the population with permeabilized mitochondria or not (Fig. 5I).

**Fig. 6:**
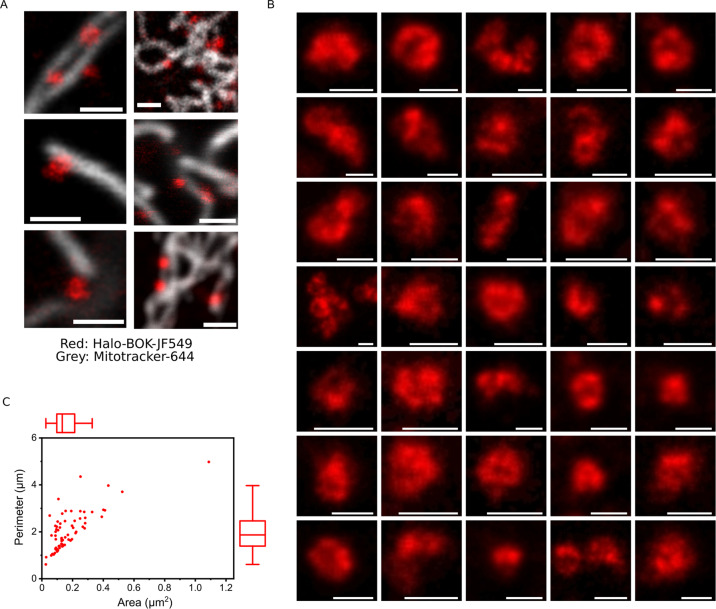
BOK forms ring-like structures in apoptotic mitochondria. **A** Representative images of Halo-BOK-JF549 16 h after transfection in U2OS BAX^−/−^/BAK^−/−^ cells, localized to mitochondria (stained with Mitotracker) acquired by STED microscopy. Scale bar, 1 µm. **B** A gallery of the structures formed by BOK, acquired by STED microscopy. Scale bar, 400 nm. **C** Summary of the structures formed by BOK. Perimeter is plotted against Area after image binarization.

We then used super-resolution STED microscopy to image the assemblies of Halo-BOK in the mitochondria of cells that had undergone Smac release (Figs. 5I and 6A). As shown in (Fig. 6b), we found that BOK organized into a mixture of structures, among which, in addition to dots, we could identify rings, arcs and lines. The resolved structures have a mean area around 0.2 µm^2^, which corresponds to an average diameter of 0.5 µm assuming a circular shape (Fig. 6C). Remarkably, these BOK assemblies were similar to those observed for BAX and BAK involved in mitochondrial permeabilization, suggesting that all three proteins follow a similar mechanism to permeabilize mitochondria.

## Discussion

Even after more than 20 years of its discovery, the exact role of BOK in apoptosis continues to be puzzling [7, 42]. A body of evidence has now accumulated supporting the classification of BOK as an effector of the BCL-2 family. Here, we provide direct visualization of BOK pores and compare their properties to those of BAX and BAK.

Using in vitro and in cell experiments, we found that BOK can induce membrane permeabilization independent of BAX and BAK. While the C-terminal anchor involved in membrane targeting was not necessary for membrane permeabilization, it contributed to protein thermostability. A possible explanation to the increased thermostability of FL-BOK is the formation of domain-swaped dimers that were shown before to inhibit BAX [43]. It is generally accepted that BAX and BAK can be activated by cBID to form pores in cells and artificial membranes [33], while this has remained less clear in the case of BOK [10, 16]. Yet, in vitro, both BAX and BAK, as well as FL-BOK shown here, can be activated by temperature, without the need for direct activators [29, 44]. This effect of temperature might be explained by promoting protein unfolding and exposure of helices involved in interaction with the membrane, which then would drive membrane insertion and pore formation. The lack of the C-terminal helix of BOK could cause decreased stability, manifested in lower melting temperature and pore activity at room temperature.

BOK has been considered a pro-apoptotic member of the BCL-2 family based on the structural homology with both BAX and BAK and on genetic studies [15]. While the majority of apoptotic stimuli cannot induce apoptosis in cell lacking BAX and BAK [41, 42], apoptosis could be triggered in these cell lines by proteasome inhibitors which correlated with BOK stabilization [13]. Accordingly, we found that overexpression of GFP-BOK induced apoptosis not only in cells knock out for both BAX and BAK, but also in cells lacking the most relevant BCL-2 family members. This indicates that the pro-apoptotic activity of BOK in cells not only does not require BAX and BAK, but also discards any potential mechanism involving a phenotypic switch of anti-apoptotic BCL-2 proteins. Since the extent of BOK-induced cell death was not affected by the presence pro-survival family members, they cannot inhibit BOK pores either.

We could directly visualize BOK-induced pores in liposomes using EM, whose features suggest that they are of toroidal (or lipidic) nature, as proposed for BAX and BAK [45]. Protein density could not be located at the pore edge [40] and the pore size was flexible and increased with protein density on the membrane. Also in agreement with a BAX-like toroidal pore, the single-molecule analysis of BOK stoichiometry revealed that BOK existed in membranes as a mixture of oligomeric species that grew into larger assemblies with protein density in the membrane.

Importantly, we were able to detect ring-shaped nanostructures of BOK directly in the mitochondria of apoptotic cells with a similar organization to those found for BAX and BAK using super-resolution microscopy [35, 46]. This suggests that BOK induces mitochondrial permeabilization in cells via a common molecular mechanism with the effector BCL-2 proteins, even if its regulation differs from that of BAX and BAK.

Also in contrast to BAX and BAK, overexpressed BOK accumulated at discrete sites co-localizing with mitochondria, the ER and likely other cellular membranes. Considering that mitochondrial permeabilization requires localization to this organelle, we propose that limited BOK localization to mitochondria is a key mechanism to control BOK pore formation and apoptosis induction. We provide evidence supporting this model by using chimeras of BOK with the C-terminal of BCL-xL, which efficiently target to mitochondria and become as potent as BAX in inducing cell death. According to this model, BOK-mediated apoptosis would be regulated by its mitochondrial accumulation, for example via upregulation via proteosomal degradation as reported by Llambi et al. in [13], or by alternative mechanisms controlling BOK localization that remain to be explored. A recent study reported that BOK levels were upregulated by the SARS-CoV-2 membrane protein (M), leading to apoptosis and lung edema in mice [47], which may open opportunities for BOK targeted interventions against COVID-19.

In summary, here we provide direct visualization of BOK membrane pores that share mechanistic properties with the pores formed by the apoptotic effectors BAX and BAK. We also show that the regulation of BOK permeabilizing activity is however different from BAX and BAK, and independent of other BCL-2 proteins. Our data support a model in which BOK apoptotic activity is controlled by subcellular localization and membrane lipid composition.

## Materials and methods

### Recombinant protein expression and purification

BOK gene (Full length (FL-BOK) and a truncated version lacking the last 24 residues (BOK∆C)) were cloned into a pETM-11 plasmid (EMBL), containing an N-terminal 6xHis tag. To enable fluorescent labeling of BOK∆C using sortase A enzyme, three glycine residues were added to the N-terminus of the protein (^3G^BOK∆C) and the gene was cloned into a pTYB21 plasmid (New England Biolabs), containing an N-terminal intein tag. BL21-(DE3)-RIPL *E. coli* (Agilent Technologies) were transformed with the expression plasmids, and was grown in LB media containing the corresponding resistance antibiotics. Protein expression was induced at OD = 1, using 1 mM isopropyl-β-D-thiogalactoside for 4 h at 18 °C. The bacteria were then retrieved by centrifugation, resuspended in Lysis buffer (20 mM Tris, 1 M NaCl, 1 mM PMSF, pH 8) and were then lysed by sonication. Afterwards, the lysate was cleared by centrifugation at 20,000 g for 1 h. The supernatant of 6xHis tag fusion proteins were incubated with Ni-NTA agarose beads (Qiagen) and eluted with 250 mM imidazole, While the supernatant of intein-tagged ^3G^BOK∆C was incubated with chitin beads (New England Biolabs) and eluted by incubation with 50 mM DTT for 40 h at 4 °C. The eluted fractions were then concentrated and injected on a Superedx 75 Increase 10/300 GL Size exclusion column (Cytiva). Protein purity was examined by SDS–polyacrylamide gel electrophoresis. Protein quantification was performed using Nanodrop and protein identity was further confirmed with Mass spectrometry. Cleaved Bid (cBID) and BAX were produced as previously described [18, 21].

### Protein fluorescent labeling

^3G^BOK∆C was fluorescently labeled using sortase A enzyme and an Atto488-conjugated peptide containing the sortase-recognition motif (LPRTG) [48]. This results in the conjugation of the dye only to the N-terminus of the protein, which is crucial for the accurate estimation of the stoichiometry of the protein. ^3G^BOK∆C was incubated with sortase A (0.3 molar equivalent) and Atto488-peptide (20 molar equivalent) in sortase buffer (20 mM Tris, 150 mM NaCl, 5 mM CaCl_2_, pH 7) at room temperature for 30 min. After that, the reaction mixture was passed through a desalting column (PD10, 25 G, GE healthcare) to remove unreacted peptide. Fractions containing protein were combined and then incubated with Ni-NTA agarose beads (Qiagen) for 30 min to remove the His-tagged sortase A. The labeled protein was then aliquoted and stored at −80 ^o^C.

### Liposome permeabilization assay

Lipids were purchased from Avanti Polar Lipids, and were dissolved in chloroform and mixed with the desired molar ratios. Chloroform was then evaporated under vacuum for 3 h. Large unilamellar vesicles (LUVs) were prepared using the extrusion method as described before [49, 50]. Briefly, the lipid film was hydrated with 80 mM solution of the fluorescent dye calcein, pH 7 for a final lipid concentration of 5 mg/mL followed by five cycles of freezing and thawing. The lipid solution was then extruded through a polycarbonate membrane with a pore size of 100 nm using glass syringes. Calcein-loaded LUVs were separated from free calcein using Sephadex-G50 beads and the lipid concentration was adjusted to 100 μM. LUVs were incubated with serial dilutions of the recombinant proteins in a 96-well plate and calcein release was monitored by fluorescence emission at 520 nm with excitation at 490 nm for 1 h using a microplate reader (Enspire, PerkinElmer). 0.1% Triton X-100 was used as a positive control (100% calcein release) and the percentage of calcein release was calculated as follows:

$${{{{{{{\mathrm{\% }}}}}}}}Calcein\,release = 100 \times \frac{{\left[ {F_{Sample} - F_{Buffer}} \right]}}{{\left[ {F_{Triton} - F_{Buffer}} \right]}}$$

### Thermal shift assay

BOK∆C, FL-BOK and BAX were diluted to 4 μM in PBS (pH 7.4) in a total reaction volume of 20 μL with SYPRO Orange (Sigma-Aldrich) used as a probe. The reaction mixture was dispensed in Microamp Fast Optical 96-well reaction plates (ThermoFisher) and analyzed in a C1000 thermal cycler (Bio-Rad). Protein melting temperature was estimated from the first derivative of fluorescence emission with respect to temperature.

### Negative staining electron microscopy

LUVs (PC:CL 8:2) were incubated with BOK∆C, FL-BOK, BAX (+40 nM cBID) and GSDMD (+20 nM caspase11) for 40 min. The protein:lipid molar ratio was adjusted to 1:100 or 1:10000. Afterwards, the proteoliposomes were placed onto a glow-discharged copper grid (Electron Microscopy Sciences) coated with a layer of thin carbon, washed twice with water, stained with 2% uranyl acetate for 5 min and then air-dried. The grids were imaged on a JEOL JEM2100PLUS electron microscope and recorded with a GATAN OneView camera (CECAD imaging Facility).

### Supported lipid bilayers (SLBs)

For the formation of LUVs, lipid mixtures were rehydrated to a final concentration of 10 mg/mL in PBS, pH 7.4. 10 μL of the multilamellar vesicle suspension was then diluted in 140 μL of SLB buffer (10 mM HEPES, 150 mM NaCl, pH 7.4). The suspension was then subjected to five cycles of freezing-thawing and was then extruded through a polycarbonate membrane with 100 nm pore size using a glass syringe. Atto488-BOK∆C was first incubated at different concentrations with LUVs made of PC:CL (8:2) for 40 min at room temperature to form proteoliposomes. The proteoliposomes were then diluted with empty liposomes (to be in the single-molecule regime) and were incubated at 37 °C for 2 min with 3 mM CaCl_2_ on plasma-cleaned glass slides (0.15 mm thickness). Afterwards, the SLB was rinsed several times with SLB buffer to remove non-fused vesicles. The SLB was then immediately imaged with TIRF microscopy. At least 500 particles were detected and analyzed per replica in each experiment.

### TIRF Microscopy

SLBs were imaged using a modified Zeiss Axiovert 20 M epifluorescence microscope with a 488 nm laser equipped with a 100x/1.46 oil objective (Zeiss), a Laser-TIRF 3 Imaging System (Zeiss) and an EM-CCD camera (iXon 897, Andor). Exposure time was set to 35 ms with a delay time between frames of 25 ms and an intensity of 0.1 kW/cm2. The images acquired were used for the stoichiometry analysis based on the fluorescence intensity of the particles using an in-house algorithm implemented in Python.

### Stoichiometry analysis

The images acquired were used for the stoichiometry analysis based on the fluorescence intensity of the particles. Bright spots were detected using the difference of Gaussians method and thresholding and were then fitted to two-dimensional (2D) Gaussians and the background was subtracted. Localized particles were filtered based on the distance and on the width of the 2D Gaussian, to avoid overlapping regions of interest (ROIs) or multiple particles in the same ROI. Brightness value for each particle was calculated from the area under the 2D Gaussian. The brightness of particles was used to decipher the stoichiometry of the proteins as previously described [28, 35]. Briefly, Atto488-BOK∆C was spread on a glass slide and individual particles with a single photobleaching step were detected and were fitted to a Gaussian to estimate the mean intensity (μ) and standard deviation (σ) of a monomer. Then, the mean brightness of different oligomers (N) was calculated from the equation: $\mu _N = N{\mu _1} \pm \sigma _1\sqrt N$. The number of Gaussians that can be fitted to the distribution of fluorescence intensity was estimated according to equation [51]: $N_{max} = \left( {\mu _1 / \sigma _1} \right)^2$.

### Mitochondrial isolation and cytochrome c release assay

DKO HCT 116 and AKO HCT116 cells were harvested and resuspended in MB buffer (10 mm HEPES, 210 mm mannitol, 70 mm sucrose, 1 mm EDTA, pH 7.5) supplemented with Complete Protease Inhibitor Cocktail (Roche). Afterwards, the cells were disrupted by passing 20 times through a 27 G needle, and the debris was removed by centrifugation at 1500 g. Mitochondria were then obtained by centrifugation at 7000 g for 10 min. The pellet was then resuspended in MB buffer and protein concentration was determined by Bradford assay (Biorad). For the cytochrome c release assays, 30 μg of mitochondria was incubated with BOK∆C, BAX and cBID in RB buffer (10 mM HEPES, 125 mM KCl, 0.5 mM EGTA, pH 7.4) for 30 min at 37 °C. The samples were centrifuged for 10 min at 14,000 g and the presence of cytochrome c in the pellet and supernatant was determined by western blotting using a mouse monoclonal anti-cytochrome c antibody clone 7H8.2C12 (BD-Biosciences). Anti-TOM22 antibody (#:sc-101286, Santa Cruz) was used as a control.

### Mammalian cell culture and transfection

HCT116 cells (WT, BAX^−/−^BAK^−/−^ (DKO), kindly provided by Prof. Schulze-Ohsthoff, and all Bcl-2 proteins knock out (AKO)) were used in the quantification of cell death activity using IncuCyte (Sartorius) and were grown in McCoy’s 5 A modified medium (Sigma-Aldrich). Caspase-9 CRISPR/Cas9 knock out were generated in HCT116 WT cells. For CRISPR transfection, 1–2 × 105 cells were seeded in a 6-well plate 48 h before transfection. 500 ng of CRISPR construct was transfected with 1 µL of Lipofectamine 2000 (Thermo Fisher) according to manufacturer’s instructions. 24 h after transfection cells were transferred to a 15 cm dish and selected for seven days with media supplemented with 0.5 µg/mL puromycin. Single colonies were picked and cultured for validation. Success of the knock out was validated using Western blotting and genotyping by Sanger’s sequencing of the target region. The following guide RNA sequence were used for the generation of CASPASE-9 CRISPR/Cas9 knock out cell lines: CAACTTCTCACAGTCGATGTTtgg. Pairs of oligonucleotides containing the gRNA sequence were cloned into the pSpCas9(BB)-2A-Puro V2.0 (px459, Addgene #62988) using the restriction enzyme BbsI (NEB). U2OS BAX^−/−^BAK^−/−^ cells were used for TMRE assay and for confocal and STED microscopy and were grown in low-glucose Dulbecco’s modified Eagle’s medium (DMEM) (Sigma-Aldrich). All media were supplemented with 10% fetal bovine serum (FBS) and 1% penicillin-streptomycin (Thermo Fisher). Cells were transfected with plasmid DNA using Lipofectamine 2000 (Invitrogen) according to the manufacturer’s protocol.

### IncuCyte cell death assays

Cell death assays were performed using IncuCyte S3 (Sartorius) at 37 °C 5% CO_2_. Apoptotic cell death was measured by the binding of Annexin V Alexa 647 (Invitrogen) to cells, which indicates the exposure of Phosphatidylserine. HCT116 cells were seeded (5000 cells/well) in a 96 well plate for 24–48 h and then transfected with 25 ng/well of plasmid DNA using lipofectamine 2000 (Invitrogen) according to the manufacturer’s protocol. Prior to transfection, the medium was replaced with medium containing 1:200 Annexin V Alexa. Four images per well were acquired every 1 h for 24 h. The images were then analyzed using IncuCyte basic analysis software module.

### TMRE Mitochondrial Membrane Potential Assay

U2OS BAX^−/−^BAK^−/−^ cells were seeded (1000 cells/well) in a 48-well plate for 24–48 h and then transfected with 50 ng/well of plasmid DNA using lipofectamine 2000 (Invitrogen) according to the manufacturer’s protocol. Prior to transfection, the medium was replaced with medium containing 100 nM TMRE dye (Invitrogen). Live cell imaging was performed using IncuCyte S3 (Sartorius) as described above. Nine images per well were aquired every 1 h for 24 h. The images were analyzed using IncuCyte Cell-by-Cell analysis software module. TMRE signal was calculated from the integrated intensity in each cell.

### Confocal and STED microscopy

For Confocal imaging, U2OS BAX^−/−^BAK^−/−^ cells seeded on coverslips were transfected with (Halo-BOK, GFP-BOK or GFPBOK∆C) and (GFPSEC61 or mCherry-SEC61) and then treated with 10 µM QVD. 16 h after transfection, the cells were incubated with 150 nM MitoTracker Deep Red (Thermo Fisher) and HaloTag TMR Ligand (Promega) (when transfecting with Halo-BOK) for 20 min at 37 °C. The cells were then washed 3 times with fresh media and were fixed using Paraformaldehyde. Imaging was performed on a TCS SP8 confocal laser scanning microscope (Leica Microsystems) equipped with a PL Apo 63x/1.40 Oil CS2 objective and a tunable white light laser (470–670 nm). The signal was acquired with sensitive HyD detectors (Leica Microsystems).

For STED imaging, U2OS BAX^−/−^BAK^−/−^ cells seeded on coverslips were transfected with Halo-BOK and Smac-GFP and then treated with 10 µM QVD. 16 h after transfection, the cells were incubated with 0.3 μM Janelia Fluor 549 HaloTag Ligand (Promega) and 150 nM MitoTracker Deep Red (Thermo Fisher) for 20 min at 37 °C. The cells were then washed 3 times with fresh media and were fixed using Paraformaldehyde. Images were acquired using TCS SP8 gSTED microscope (Leica Microsystems) equiped with HL PL APO 100x/1.40 Oil STED, a tunable white light laser (470–670 nm) and 750 nm depletion laser. The signal was acquired with sensitive HyD detectors (Leica Microsystems).

## Supplementary information

Supplementary material

uncroppred WB figure 4

checklist

new author inclusion form

## Data Availability

The data that support the findings of this study are included in the article or uploaded as Supplementary Information and all original data are available from the corresponding author upon request.
