# Supplementary material for: Visualization of BOK pores independent of BAX and BAK reveals a similar mechanism with differing regulation
Source: Cell Death Differ. 2022 Oct 26;30(3):731–41. doi: 10.1038/s41418-022-01078-w (PMC9607731; doi:10.1038/s41418-022-01078-w)
Supplement: Supplementary file 1 — Supplementary material [file 41418_2022_1078_MOESM1_ESM.pdf]

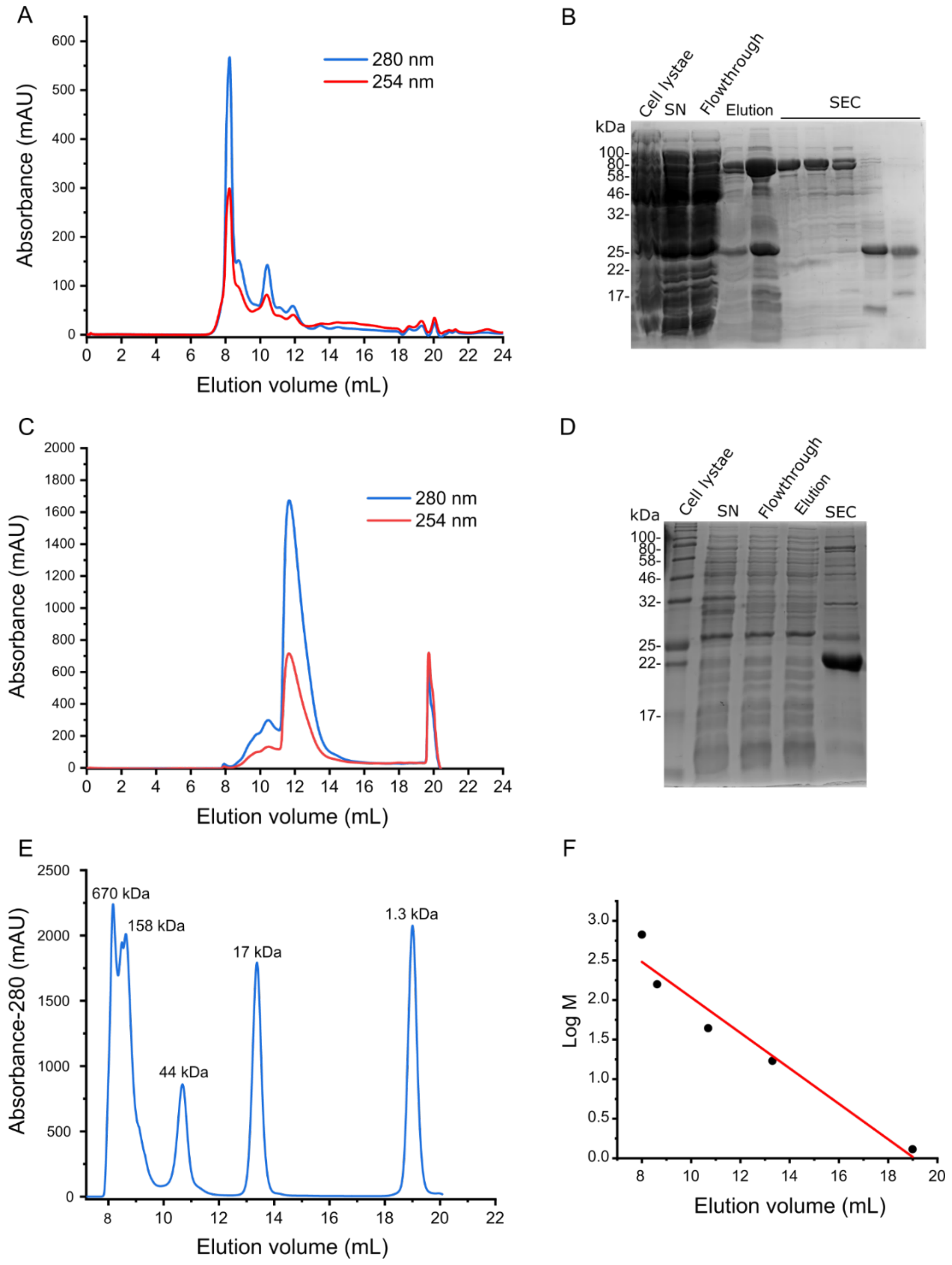

Figure S1. Purification of recombinant BOK∆C and FL-BOK

(A) SEC Chromatogram from Superdex75 column after injection of the elution fraction of FL-BOK (B) Representative Coomassie-stained SDS-PAGE gel for the steps of the purification process of recombinant FL-BOK. SN: supernatant after lysis and centrifugation, SEC: size-exclusion chromatography for fractions from 7 to 11 mL. (C,D): The same as in (A,B) but for the purification of BOK∆C. (SEC fraction in the SDS-PAGE corresponds to the peak with maxima at 12 mL). (E) SEC Chromatogram from Superdex75 column using a mixture of proteins with different molecular weights (Gel Filtration Standard, Bio-rad #1511901). (F) Calibration curve of Superdex75 column derived from elution profile in (E).

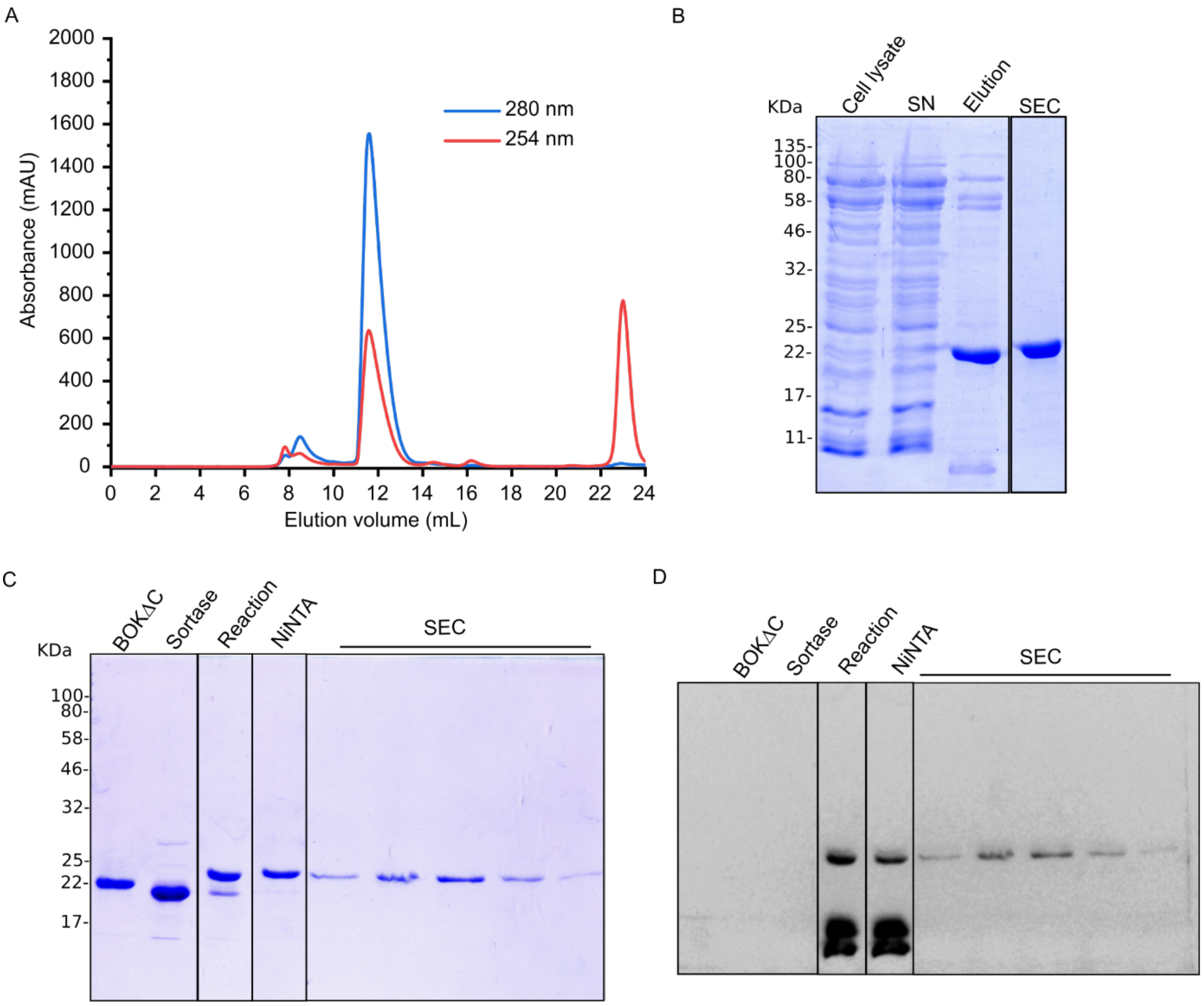

Figure S2. Purification and fluorescent labelling of recombinant 3G-BOKΔC

(A) Size-exclusion chromatogram from Superdex75 column after injection of the elution fraction of ^{3G}BOKΔC showing a dominant monomeric peak (B) Representative Coomassie-stained SDS-PAGE gel for the steps of the purification process of recombinant ^{3G}BOKΔC. SN: supernatant after lysis and centrifugation, SEC: size-exclusion chromatography (SEC fraction in the SDS-PAGE corresponds to the peak with maxima at 12 mL). (C,D): Representative SDS-PAGE gel for the steps of the fluorescent labelling of 3G-BOKΔC using an ATTO488-linked peptide and SortaseA enzyme. The same gel was Coomassie-stained (C) and visualized under UV irradiation (D). NiNTA: a fraction from the reaction mixture after incubation with NiNTA beads, SEC: size-exclusion chromatography using G25 desalting column.

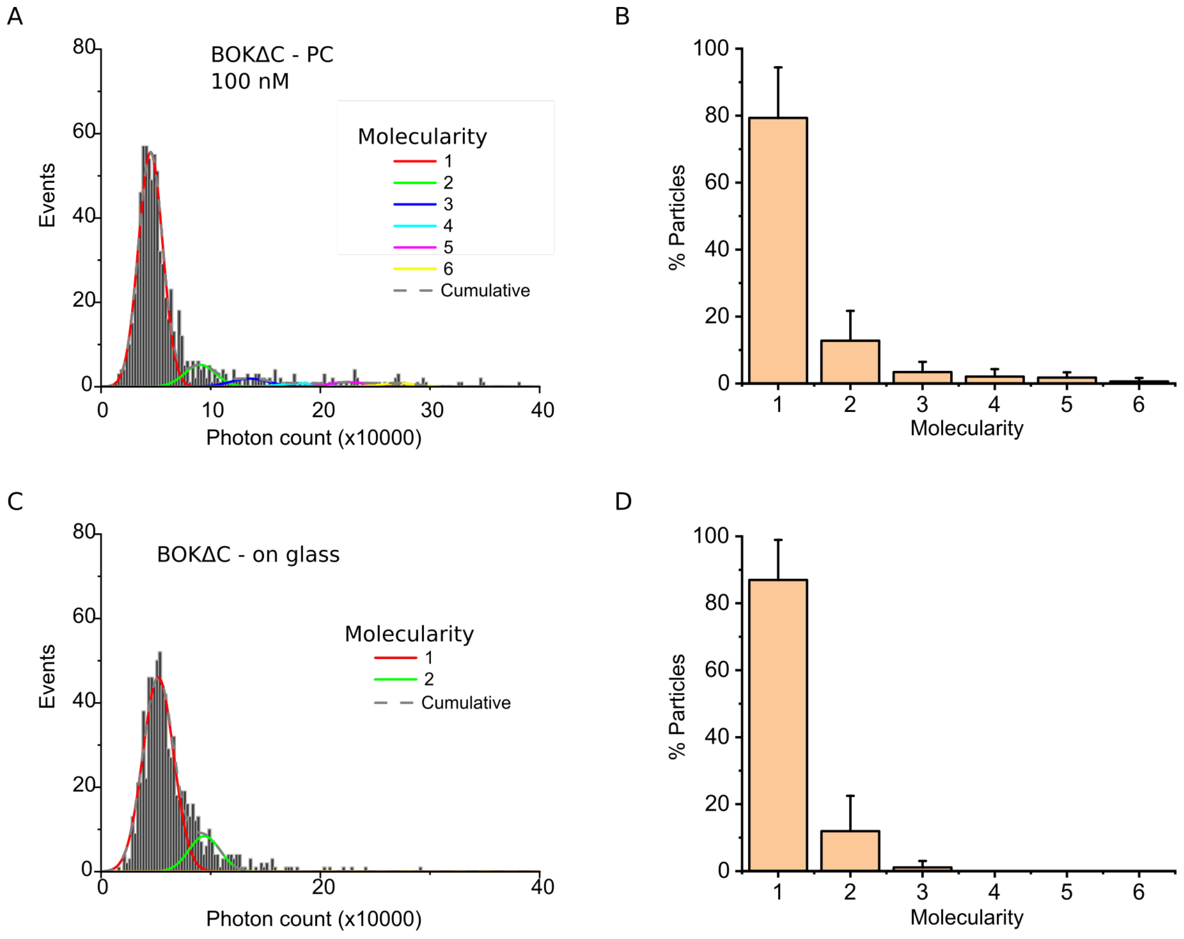

Figure S3. Characterization of BOKΔC oligomerization in SLB made from PC and on glass

(A,C) Particle fluorescence intensity distribution of BOKΔC-488 from different experiments were fitted with a linear combination of six Gaussians to estimate the abundance of different molecularities. The cumulative fit is shown as a dashed line. (B,D) The percentage of each species is derived from the area under each fitted Gaussian. The error bars correspond to the average error for each oligomeric species from three independent experiments with Particles>500 per condition per experiment.

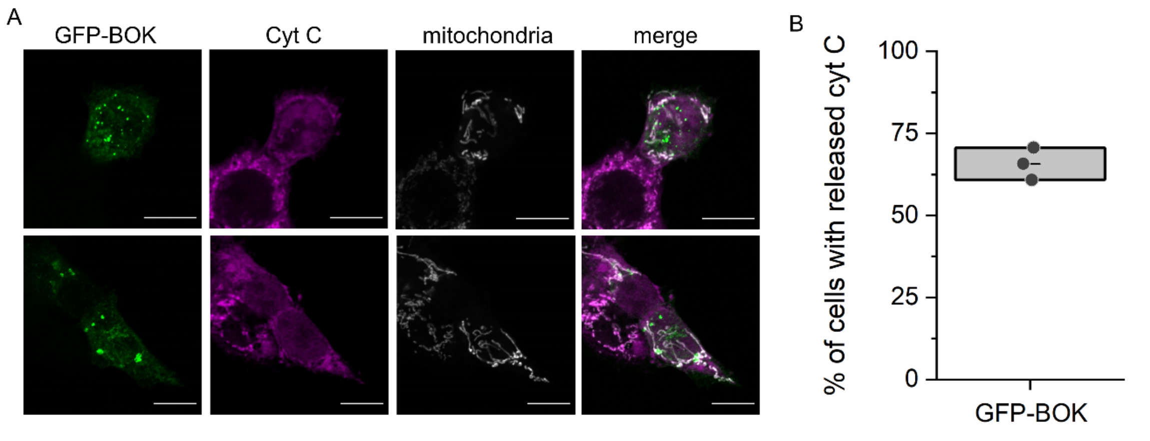

Figure S4. GFP-BOK overexpression induces cytochrome c release in HCT AKO cells.

(A) Representative confocal immunofluorescence images of subcellular localization of GFP-BOK (in green), mitochondria (grey) and cytochrome c (magenta). HCT AKO cells were transiently transfected with a GFP-BOK encoding plasmid using lipofectamine 2000 (Life Technologies) and incubated for 18–24 h. Cells were incubated with 200 nM MitoTracker 561 at 37 °C for 30 min and fixed with 3.8% paraformaldehyde in PBS. Samples were blocked with 3% bovine serum albumin (BSA)/0,1% Triton-X100 in PBS and immunoblotted with a primary anti-cyt c anti-body (1:200, BD-556432) and a secondary fluorescent anti-mouse 633 antibody (1:400, Life Technologies A-21126). Scale bars 10 μm. (B) Effect of GFP-BOK on MOMP, measured as percentage of cells showing released cyt c. Data correspond to three independent experiments, n>15 cells per condition per experiment.
