# Supplementary figures and images for: Visualization of BOK pores independent of BAX and BAK reveals a similar mechanism with differing regulation
Source: Cell Death Differ. 2022 Oct 26;30(3):731–41. doi: 10.1038/s41418-022-01078-w (PMC9607731; doi:10.1038/s41418-022-01078-w)

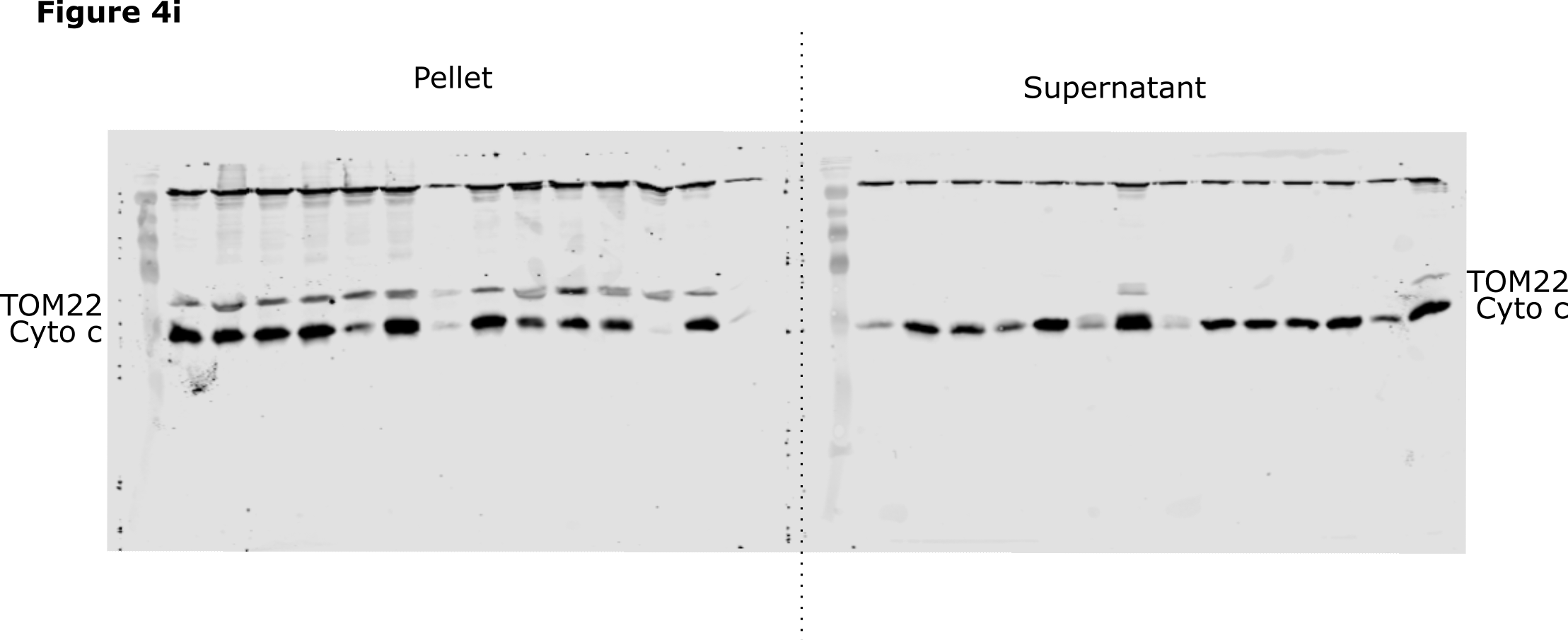

Supplement: Supplementary file 2 — uncroppred WB figure 4 [file 41418_2022_1078_MOESM2_ESM.png]
